# *Trichuris* spp. infecting domestic cats on St. Kitts: identification based on size or vulvar structure?

**DOI:** 10.1186/s40064-015-0892-z

**Published:** 2015-03-05

**Authors:** Jennifer K Ketzis

**Affiliations:** Department of Biomedical Sciences, Ross University School of Veterinary Medicine, PO Box 334, Basseterre, Saint Kitts and Nevis

**Keywords:** Cats, *Trichuris* spp, *Trichuris serrata*, *Trichuris campanula*

## Abstract

**Background:**

On St. Kitts, a high number of cats are found to be infected with *Trichuris*. Necropsies have shown pathologic changes related to the infections. In order to determine if these changes were related to a particular *Trichuris* species, a review of the original identifications of *Trichuris campanula* and *Trichuris serrata* was conducted.

**Findings:**

Based on the review of published descriptions of *T. campanula* and *T. serrata*, it is hypothesized that the presence or absence of a vulvar projection and a bacillary band can be used to differentiate the two species and these criteria are more accurate than nematode or egg size. The *Trichuris* in cats on St. Kitts were similar in size to the description of *T. campanula*, but had both a vulvar projection and bacillary band.

**Conclusions:**

Based on the morphological characteristics, all of the *Trichuris* found in twelve domestic cats were determined to be *T. serrata*. Cats without pathologic changes are required to further investigate if the changes are species or strain related.

## Introduction

Two species of *Trichuris* in domestic cats, *Trichuris serrata* and *Trichuris campanula*, were originally described by von Linstow 1879, 1889, respectively. The number of worms used in these original descriptions, isolated from cats in Brazil, was limited and no whole male was available for *T. campanula*. In 1923, Urioste described specimens from Brazil and asserted that there was only one species infecting cats, *T. serrata* (Cameron 1937; Clarkson and Owen 1960). However, his description was later combined with von Linstow’s original description of *T. campanula* to provide a more complete description of the species (Clarkson and Owen 1960; Ng and Kelly 1975). Descriptions of the two species were expanded by Cameron (1937), Clarkson and Owen (1960), Kelly (1973) and Ng and Kelly (1975). Neither of these species has been described using molecular methods.

There is some debate regarding if there really are two species of *Trichuris* in cats or just one species (Bowman et al. 2002). However, according to the original descriptions by von Linstow (1879 and 1889), a description of *T. serrata* by Cameron (1937) and descriptions of both species by Kelly (1973) and Ng and Kelly (1975), the distinguishing characteristic between the species is the presence of a finger-like projection on the vulva of *T. serrata* versus two inconspicuous longitudinal lips on the vulva of *T. campanula*. In addition, Ng and Kelly (1975) used the absence of a bacillary band in *T. campanula* to distinguish it from *T. serrata*. The eggs, in all of these identifications, have been described as typical Trichuroid (light brown, lemon shaped with biopolar plugs and a morula) with no particular distinguishing morphological characteristics.

In other identifications, size of the adult worms and the eggs have been used to distinguish the two species, with *T. campanula* adult worms being slightly smaller and the eggs being slightly larger than those of *T. serrata*. When Clarkson and Owen (1959) first described *Trichuris* from cats in the Bahamas, they described the size as that of *T. campanula* and the finger-like projection as that of *T. serrata*. An archived specimen of *T. campanula* was obtained from Dr. G. Hatwich of the Zoological Museum in Berlin (no *T. serrata* specimens were available) to assist Clarkson and Owen (1960) in their identification. The archived specimen also had the finger-like projection, and therefore, the Bahaman species was considered *T. campanula*. Based on this, both *T. campanula* and *T. serrata* have the projection and size is the distinguishing characteristic. There was no mention regarding the presence or absence of bacillary bands in either of the specimens (that from the Bahamas or that from Berlin). Also, the measurements of the Berlin specimen are not included in the description by Clarkson and Owen (1960).

Since the identification by Clarkson and Owen (1960), feline *Trichuris* identification often has been based on egg or adult size, by referencing the description provided by Clarkson and Owen, and with no mention in regard to a vulvar projection or the bacillary band (Hass and Meisels 1978; Beldomenico et al. 2005). The exception to this is the identification by Kelly (1973) and Ng and Kelly (1975) who, as previously stated, relied on the presence or absence of the vulvar projection and the bacillary band for identification. A summary of the morphological characteristics of the two species are presented in Table 1.

**Table 1:** **Morphological descriptions of** ***Trichuris campanula*** **and** ***Trichuris serrata***

	***T. campanula***			***T. serrata***			**St. Kitts**
	**von Linstow (** 1889 **)**	**Ng and Kelly (** 1975 **)**	**Clarkson and Owen (** 1960 **)**	**von Linstow (** 1879 **)**	**Kelly (** 1973 **)**	**Cameron (** 1937 **)**	
Female	31.5	38	22.5	48	49.5	14.74	25
Length (mm)	23.0^*^						(14–31)
Vulva	2 not very prominent lips	Straight; 2 inconspicuous longitudinal lips	Prominent spiny structure directed posteriorly	Spiny structure directed posteriorly	Appendage on posterior lip directed posteriorly	Appendage on posterior lip directed posteriorly	Appendage on posterior lip directed posteriorly
Eggs (μm)	72 x 36	81-85 x 35-39	63 x 34	56 x 39	54 x 40	60 x 35	60-75 x 30–40 (38 eggs)
	80 x 36^*^						
Male	20.5^*^	36	21.5	40	38	12.75	24 (17–32)
Length (mm)							
Spicule	1.5^*^	2.9	1.5	3.9	3.6	1.2	1.4
(mm)							(1.1-1.5)
Other	--	No bacillary band	--	Bacillary band	Bacillary band	Bacillary band	Bacillary band
Source	Domestic cat	Domestic cat	Domestic cat	Domestic cat	Domestic cat	Ocelot	Domestic cat
	Brazil	Australia	Bahamas	Brazil	Australia	Trinidad	
Worms used	1 female	<36 total	11 females	Unknown	6 females	3 females	22 females
	1 partial male		6 males		13 males	2 males	12 males
	Unknown^*^						9 cats for eggs

^*^From Urioste 1923 as described by Clarkson and Owen (1960) and Ng and Kelly (1975).

On St. Kitts, an island in the West Indes, as many as 71% of feral cats and 22% of owned cats are infected with *Trichuris* (Krecek et al. 2010; Ketzis and Shell 2015). In necropsies performed on cats euthanized due to ill health (e.g., Feline immunodeficiency virus positive) or accidents (e.g., dog attack, hit by car), lesions related to *Trichuris* infections have been seen grossly in some of the cats and via histopathology in other cats. Some of the lesions are similar to those described by Kirkova and Dinev (2005) in *Trichuris vulpis* dog infections. Based on the literature, *T. serrata* and *T. campanula* occur in the Caribbean; therefore, either species could be the cause of the lesions seen on St. Kitts. Since species and strain differences in parasites can be related to their virulence and pathogenesis, this study was undertaken to determine the *Trichuris* species infecting cats on St. Kitts using the published morphological descriptions.

## Results and discussion

**Figure 1:**
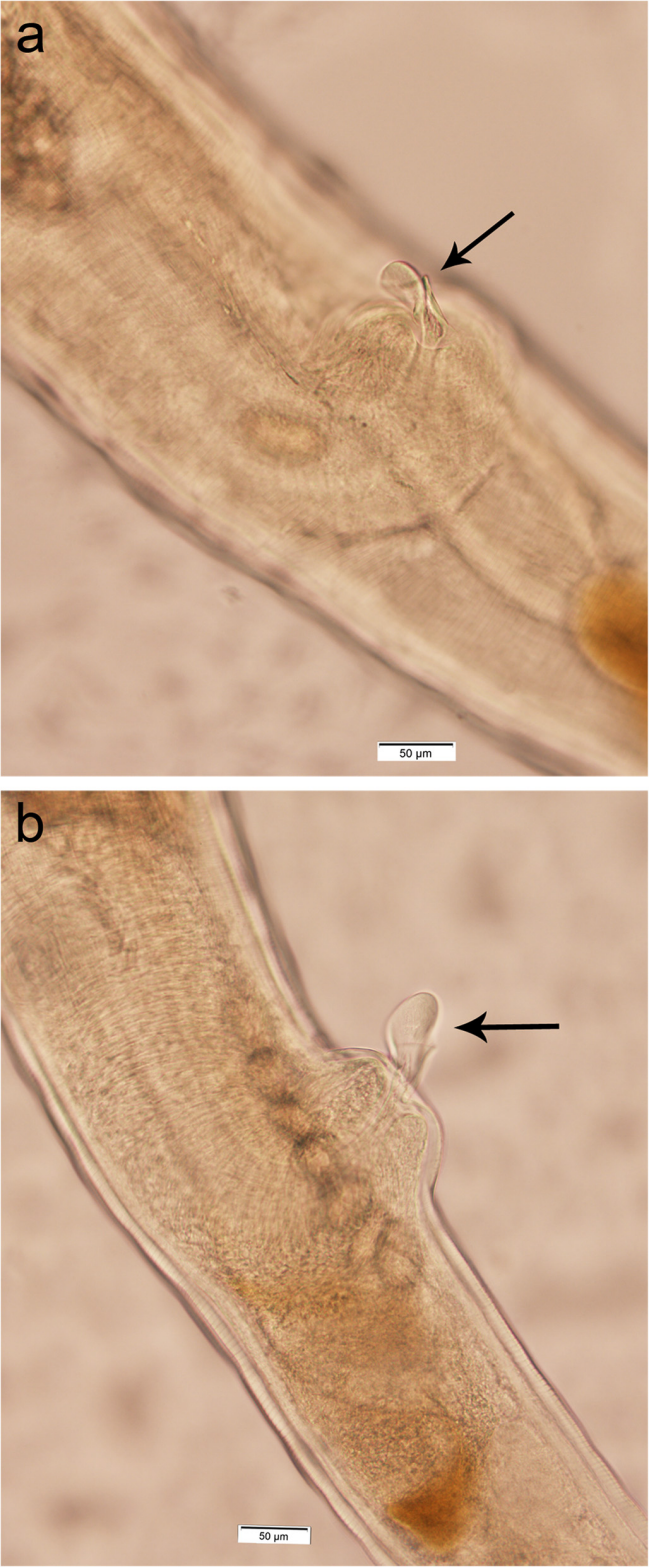
**a and b Vulvar projection (200x) of two female** ***Trichuris*** **from different St. Kitts’ cats.**

**Figure 2:**
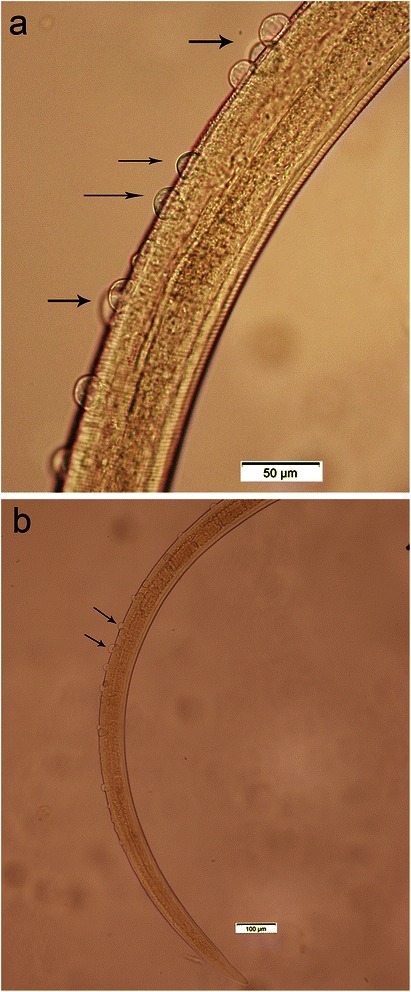
**a and b Bacillary band (100x and 200x) of two female** ***Trichuris*** **from different St. Kitts’ cats.**

**Figure 3:**
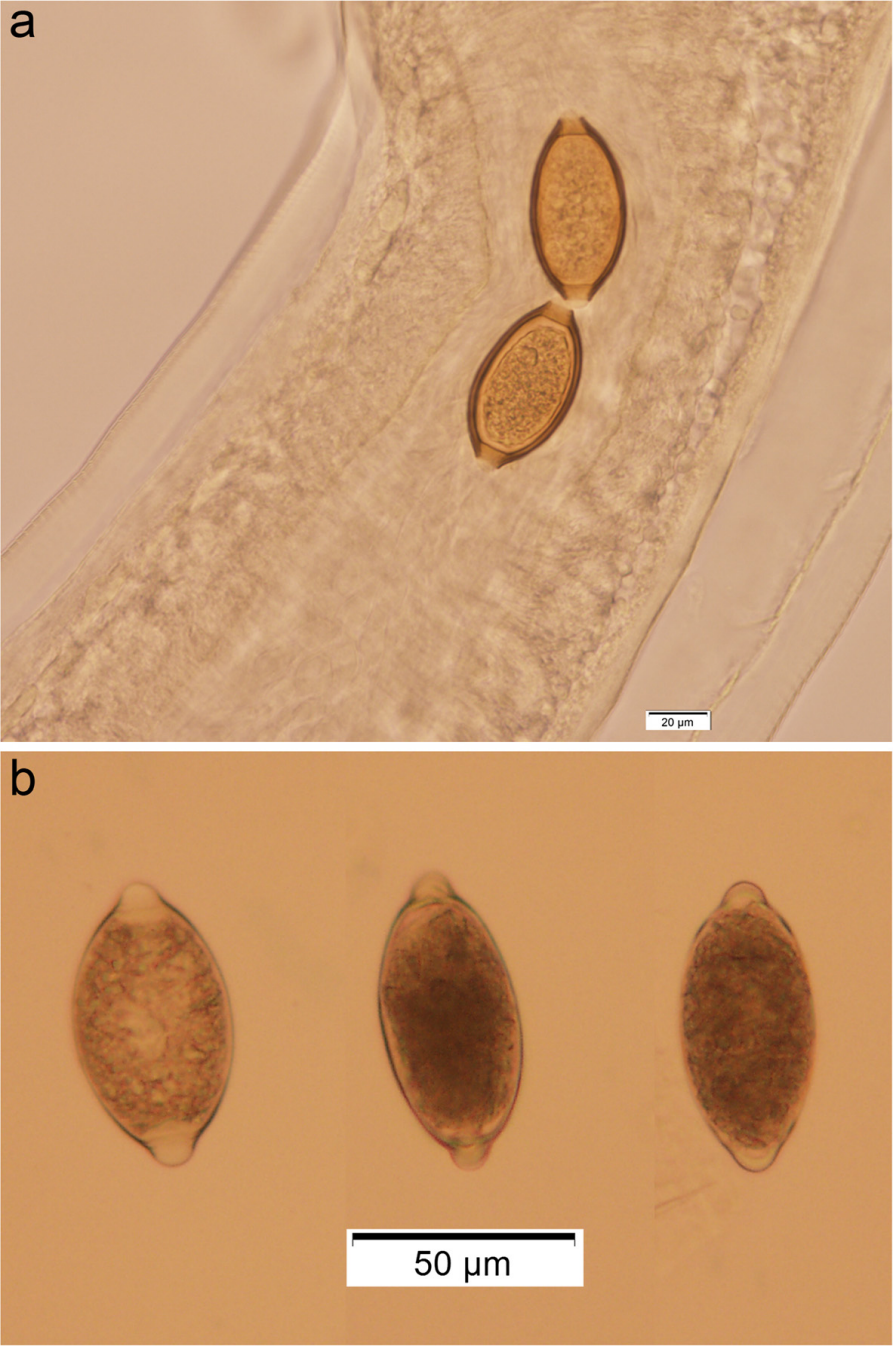
**a and b** ***Trichuris*** **eggs (100x) of different sizes seen with a fecal flotation from one St. Kitts’ cat.**

The number of *Trichuris* recovered ranged from 1 to 117 with 96 male and 113 female adults collected (Table 2). All of the female worms had a finger-like projection on the vulva and all of the worms had a bacillary band (Figures 1a and b and 2a and b). The length of the male worms (twelve measured) ranged from 17 to 32 mm and the female worms (twenty two measured) ranged from 14 to 31 mm (Table 1). The eggs ranged in size from 60–75 × 30–40 μm (38 measured; two to five per cat) and were typical Trichuroid (Figure 3a and b).

**Table 2:** ***Trichuris*** **from domestic cats on St. Kitts used for identification**

**Cat**	***Trichuris*** **recovered**			***Trichuris*** **for identification**		
	**Total**	**Males**	**Females**	**Males**	**Females**	**Eggs**
1	29	16	13	0	1	--
2	6^*^	4	2	4	1	5
3	5^*^	2	3	2	2	5
4	4	3	1	2	0	2
5	6^*^	3	3	3	3	3
6	117	48	69	0	1	5
7	7^*^	3	4	0	3	5
8	21	15	6	0	4	5
9	2	0	2	0	1	--
10	5^*^	1	4	1	3	3
11	1	0	1	0	1	--
12	6	1	5	0	2	5
Total	209	96	113	12	22	38

^*^Total does not include adult worms attached to intestinal sections submitted for pathology.

Based on the presence of the finger-like projection and bacillary band, the *Trichuris* in cats on St. Kitts is *T. serrata*. However, if size is used for classification, then the parasite is more likely to be *T. campanula*. The eggs, on the other hand, are similar in size to both species with none of the eggs as large as some of the descriptions for *T. campanula* nor as small as some of the descriptions for *T. serrata*.

One of the difficulties in distinguishing the two species of *Trichuris* in cats is that few whole male and female worms were used in the original descriptions by von Linstow and there are only two researchers that have described both species: Kelly (1973) with eight male and six female adult worms identified as *T. serrata* and Ng and Kelly (1975) with an unspecified number of adult male and female worms identified as *T. campanula*; and Hass and Meisels (1978) with six adult worms from Florida identified as *T. campanula* and one non-intact male from Wisconsin identified as *T. serrata*. Ng and Kelly used the von Linstow (1879 and 1889) descriptions and Hass and Meisels (1978) used Clarkson and Owen (1960) as the reference for species identification.

Another difficulty in identification of the species is the description of *T. campanula* provided by Clarkson and Owen (1960). While this is the only description of *T. campanula* with a finger-like vulvar projection, this description was instrumental in distinguishing the parasites by size. The discrepancy created by Clarkson and Owen might be due to the projection not being seen in the originally identified *T. campanula* (i.e., both species have it), an omission in the first description of *T. campanula* since it was described after *T. serrata* or the archived specimen used might have been mislabeled. The drawings provided by Clarkson and Owen almost perfectly match those of Cameron’s *T. serrata* and the description provided by von Linstow of *T. serrata*.

If mislabeling occurred and only *T. serrata* has a projection, this does not address the issue of the size difference between Clarkson and Owen’s specimens and those of other *T. serrata* specimens. While the *T. serrata* described by Cameron (1937) also was quite small and Ng and Kelly state that size is not definitive, these adult *Trichuris* are much smaller than the original description by von Linstow. Explanations for the size difference include: natural variation; a high larval exposure resulting in smaller adults; repeat infections in the host with host immunity decreasing adult worm size; and the number of adult worms present with population density regulating size (McKellar 1993; Stear et al. 1997; Walker et al. 2009; Luong et al. 2011). All of these effects have been seen in different gastro-intestinal species with variations in adult size being specifically noted with *Trichuris muris* and *Trichuris suis* (Michael and Bundy 1989; Kuchai et al. 2013). If these explanations are correct, they support Ng and Kelly’s statement that the use of the existence or absence of the finger-like projection on the females is a better means of distinguishing the species versus using parasite size.

Frequently egg size is used for identification, especially in prevalence and treatment studies. However, the egg sizes overlap for the two species. Also, in other *Trichuris* species, it has been demonstrated that egg size can significantly vary between adult worms and within adult worms (Yoshikawa et al. 1989; Areekul et al. 2010). Hence, egg size does not appear to be a reliable determinant of species.

Since neither of the species has been molecularly characterized, this method could not be used to identify the species on St. Kitts. Molecular characterization will be useful for differentiating the species when specimens matching the description of *T. serrata* and *T. campanula* are available.

## Conclusion

After a review of the literature on the identification of *Trichuris* in cats, it is concluded that the species on St. Kitts is *T. serrata*. While the size more closely matches the *T. campanula* described by Clarkson and Owen (1960), it is hypothesized that the vulvar projection only occurs in *T. serrata* and parasite size is not a reliable criterion for distinguishing the species. To determine if the pathology associated with these infections on St. Kitts is species or strain related, morphological descriptions and molecular characterization of *Trichuris* from cats without pathologic changes are required; this might potentially impact the importance of treating *Trichuris* infections in cats world-wide.

## Methods

The large intestine was collected from twelve cats euthanized due to health issues between February 2013 and March 2014. The large intestine was opened, soaked in saline at approximately 36°C for 1 h and then washed over a 50 μm sieve to collect the adult *Trichuris*. All of the intact adults collected were examined for a bacillary band. All female adults were examined for a finger-like vulvar projection. One to four adult male (representing five cats) and one to four adult female worms (representing eleven cats) were morphologically examined in more detail: length of the male and female worms, length of the esophagus and length of the spicule were measured. The number selected was based primarily on the ability to separate whole undamaged worms. Lastly, eggs harvested from the female worms and eggs retrieved from the feces were measured from nine of the cats. An Olympus SZX16 with a microscope mounted camera (Olympus DP73) was used for photographing the parasites and these photographs with a stage micrometer were used for subsequent measuring of the parasites. Photographs of the vulvar projection, bacillary band and eggs were obtained using an Olympus BX41 with a microscope mounted camera (Olympus DP73). Eggs were measured using an ocular micrometer.

### Ethical standards

All procedures in regards to the decision to euthanize the cats from which the *Trichuris* were harvested were approved by the Ross University School of Veterinary Medicine Institutional Animal Care and Use Committee. All institutional and national guidelines for the care and use of laboratory and study animals were followed.
